# The status of the global food waste mitigation policies: experience and inspiration for China

**DOI:** 10.1007/s10668-023-03132-0

**Published:** 2023-04-05

**Authors:** Guohui Shen, Ziqi Li, Tiannuo Hong, Xin Ru, Kunzhen Wang, Yanting Gu, Juan Han, Yanzhi Guo

**Affiliations:** 1 Institute of Food and Nutrition Development, Ministry of Agriculture and Rural Affairs, Beijing, 100081 China; 2 Chinese Academy of Agricultural Sciences, Beijing, 100081 China

**Keywords:** Food safety, Anti-food waste, Policy, International comparison, China

## Abstract

Food waste has become a significant challenge faced by the community with a shared future for mankind, and it has also caused a considerable impact on China's food security. Scholars across disciplines, international organizations, and especially policymakers are increasingly interested in food waste. Policies are seen as a powerful factor in reducing food waste, but current research on related policies is more scattered. This paper summarizes and analyzes the experiences of food waste policy development and implementation by systematically reviewing the studies on food waste reduction policies. The results of this paper's analysis show that current global food waste policies are focused at the national strategic level, with approaches such as legislation, food donation, waste recycling, awareness and education, and data collection. At the same time, we find that the current experience of developed countries in policy formulation and implementation is beneficial for policy formulation in developing countries. And taking China as an example, we believe that developing countries can improve food waste policies in the future by improving legislation, guiding the development of food banks, promoting social governance, and strengthening scientific research projects. These policies will all contribute strongly to global environmental friendliness. In addition, we discuss some of the factors that influence the development of food waste policies and argue that in the future, more consideration needs to be given to the effects of policy implementation and that case studies should focus more on developing countries. This will contribute to the global sustainable development process.

**Supplementary Information:**

The online version contains supplementary material available at 10.1007/s10668-023-03132-0.

## Introduction

In recent years, extreme climate change and the COVID-19 pandemic have severely affected world food production (Cheng et al., 2020; Si et al., 2020; Zhong et al., 2020). Furthermore, the Russian-Ukrainian war has also affected the world food supply (Ben Hassen & El Bilali, 2022). A series of events has also further highlighted the vulnerability of agricultural food systems^1^ (Fan et al., 2022). Food waste mitigation is an essential factor and a global problem (Alamar et al., 2018; Avagyan, 2017, 2020; Morone et al., 2019b). Statistics show that the world food waste reached 931 million tons in 2019, and 17% of the world's total food production may end up wasted. In addition, compared to previous studies, food waste was high in all countries, regardless of income level. The State of Food Security and Nutrition in the World 2022 shows that the number of people affected by hunger in the world in 2021 reached 828 million, and approximately 29.3% of the world's population face moderate or even severe food insecurity (FAO et al., 2022).

Food waste significantly impacts the economy, society, and the environment (Avagyan, 2020; Garnett, 2011; Kibler et al., 2018; Liu et al., 2013). Relevant data show that the total cost of food waste is US $2.5 trillion per year worldwide^2^. Among the impacts of food waste are the inefficient use of water and land and unnecessary greenhouse gas emissions, leading to diminished natural ecosystems and the services they provide (Alvarado et al., 2021; Cattaneo et al., 2021; Jafari et al., 2020; Otles & Kartal, 2018). If global food waste were considered a country, it would be the world's third-largest emitter of greenhouse gases. Additionally, food waste will lead to the decline of biodiversity,^3^ affect farmers' income, increase the burden on low-income people, and hinder the realization of the world's poverty reduction goals. A rise in carbon emissions has a significant effect on infant mortality (Shobande, 2020). If this trend continues, SDG 12.3^4^ will be difficult to achieve. Environmental sustainability will not be achieved and hunger will persist (Shobande, 2023; Shobande & Asongu, 2022).

Currently, the academic research on food waste primarily focuses on the definition of related concepts, the measurement methods, and the data collection of food waste. A small number of scholars have conducted empirical studies related to food waste. Fewer studies have collated and compared food waste policies.

Many studies have been conducted on the concepts related to food loss and food waste. However, there is no uniform definition of food waste. In addition, the different methodological approaches adopted address different types of “loss” or “waste”, leading to results which are not comparable (Corrado & Sala, 2018; Xue et al., 2017). Table S1 summarizes the major international organizations and scholars' current definitions of food waste. It can be seen that the definition of food waste is defined by many scholars and institutions as the end of the supply chain and has a strong relationship with human behavior (Chaboud & Daviron, 2017; Parfitt et al., 2010; Zhang et al., 2019). Some scholars also believe that food waste should include the gap between the energy value of food consumed per capita and the energy value of food required per capita (Smil, 2004). Indeed, the absence of a consolidated methodological approach could undermine a deep understanding of the results from the recipient of the information (Corrado et al., 2019).

The measurement of food waste varies by stage. FAO's material flow model, research methods, and literature review methods are the more common measurements. The FAO material flow model divides the food supply chain into five segments. The five segments include production, post-harvest handling and storage, processing, distribution, and consumption. Calculation of food waste by stage using data from FAO food balance sheets (Huang & Nie, 2016; Kummu et al., 2012). The research method investigates food waste in one or more areas and at one or more points in the food supply chain and includes a variety of methods such as bookkeeping, archeological methods, weighing, and dietary review (Lu et al., 2022b). The literature review method makes judgments about the status of the food waste in response to the consolidation of the relevant research literature. The advantages and disadvantages of the various methods are compared in Table S2.

Food waste mitigation is a global problem, and many countries in the world have taken certain measures for food waste in terms of laws, systems, and policies. Policies play an important role in reducing food waste (Fesenfeld et al., 2022). Reducing food waste would contribute to addressing interconnected sustainability challenges, such as climate change, food security, and natural resource shortages. Therefore, developing an appropriate strategy for reducing FLW is one of the important issues related to sustainable development (Ishangulyyev et al., 2019). China has a tradition of food conservation since ancient times, but mostly restrains human behavior in terms of morality and habits. With modern management, including national policies, legislative system, action guidelines, and international cooperation, it still lags behind the international community, especially developed countries. Some scholars in China have reviewed the anti-food waste policies of the EU, the USA and some Asian countries (Shen, 2020; Shen et al., 2022; Yang et al., 2021; Zong, 2015). Huang Xisheng conducted a review and comparative study of overseas food conservation legislation, which provided a useful reference for China's Anti-Food Waste Law (Huang & Rao, 2021). Rosalinda Nicastro provides an overview of case studies and examples of legislation in different countries and actions taken by various players in the food chain and non-profit organizations in order to effectively prevent or reduce food loss and waste (Nicastro & Carillo, 2021a,b). The current academic review of food waste policies is primarily focused on individual countries and therefore lacks policy comparisons of the same measures in different countries.

In this study, we present a literature review of the existing scholarly discussion on the policy for reducing food waste in a systematic, transparent, and replicable way. We compared the major global policies to reduce food waste and make recommendations for improving them based on the Chinese context. We hope to systematically sort out China's food waste policies in different periods and make a comparative analysis of different countries' food waste reduction policies, so as to provide reference for developing countries to formulate food waste reduction policies and promote the realization of Sustainable Development Goals.

The contribution provided by this systematic literature review is threefold: firstly, this study will compare the major global food waste reduction policies and summarize the existing experiences; secondly, to propose recommendations to enhance the implementation of food waste reduction policies in China in the Chinese context; and finally, to advance the development of food waste reduction policies globally, especially in developing countries, and reduce the resource and environmental effects caused by food waste, thus promoting the achievement of sustainable development.

The subsequent sections of this paper are divided as follows: Sect. 2 describes the research methods used in this research; Sect. 3 analyzes the results of the study; Sect. 4 discusses the results of the study and discusses future research directions; Sect. 5 gives the research conclusions.

## Materials and methods

To interrogate the extant literature in this area in a comprehensive manner, a systematic review was conducted following Preferred Reporting Items for Systematic Reviews and Meta-Analyses (PRISMA) guidelines (Li et al., 2022; Page et al., 2021; Petticrew & Roberts, 2006). While every effort is made to ensure that the most relevant studies are selected for the SLR, it is acknowledged some factors could limit the generalization this study's findings and analysis due to selection/exclusion criteria, research design, limited.

In our study, we first positioned relevant studies based on our synthesis objective, which was to compare global policies to reduce food waste. We first searched for peer-reviewed journal articles published in English and Chinese. Also, because policy materials involve many reports and online literature, we collected some of the "gray" literature through other methods and included it in our study. In fact, this gray literature provides a lot of valuable information.

The databases Web of Science, Scopus, and CNKI were used as a basis for the literature search. The initial key word search included the search strings “food waste” AND “reducing” AND “policy” as well as “食物浪费” AND “政策” AND “国际经验” in Chinese characters. At the same time, we searched part of the content through organizations, websites and reports (Table 1). Subsequently, the articles generated from the initial search were checked manually (mainly by reading through the abstract). We excluded studies that (1) studies involving only the quantification of food waste were excluded; (2) only empirical analysis of food waste is available and does not involve policy research; and (3) those involving only food loss not food waste (Table 2).

**Table 1:** Search strategy

No.	Database	Search words	Inclusion Type	Quantity
1	Web of science	Food waste AND reducing AND policy	Article or review article	1148
2	Scopus	Food waste AND reducing AND policy	Article or review article	380
3	CNKI	食物浪费政策/国际经验	Article or review article	63
4	Website	Food waste policy	News/policy/law/commentary	10
5	Reports/books	Food waste policy	Reports/books	5
6	Organization	FAO, EU, WRAP, etc.		
				1606

**Table 2:** Exclusion criteria

No.	Exclusion criteria
1	Studies involving only the quantification of food waste were excluded
2	Only empirical analysis of food waste is available and does not involve policy research
3	Those involving only food loss not food waste

**Fig. 1:**
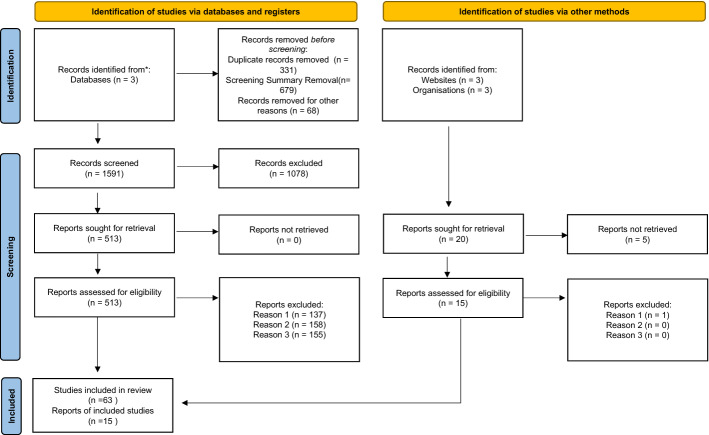
Literature search chart

According to the literature and reports we retrieved, we excluded the materials that were not relevant to our research content. A total of 78 articles were finally included in the research, including 63 literatures and 15 reports (Fig. 1). All the included information is described in the attachment.

While this paper has taken a global focus, we are more focused on how developing countries can learn from the advanced food waste reduction policies of developed countries. Therefore, in the final discussion section, we take China as an example and make corresponding suggestions based on national conditions by comparing global food waste reduction policies. This could be a potential avenue for further research on the factors that should be considered in the development of these policies and the effects of their implementation.

## Result

### National law documents

#### Focusing on food waste at a strategic national level

The initial measures, which were introduced in the International Agreement on World Food Security, were primarily aimed at food security. It was adopted by the World Food Conference in 1974 (Yang, 2001) and clearly stated the need to reduce food waste, achieve increased food consumption and reduce losses, and avoid a world food crisis. The Convention on Biological Diversity and the Rome Declaration on World Food Security, issued later, also had provisions for reducing food waste. After 2011, the United Nations began to expand the fight against food waste to the entire food chain. Several later documents have clarified the impact of food waste on the global economy, society, and the environment. The 2030 Agenda for Sustainable Development, adopted in 2015, also specifies a target to be achieved by 2030 against food waste. The EU has started to introduce a series of measures to reduce food waste in its member states after 2015 with the Circular Economy Package, including the EU Food Waste Measurement Methodology, and the Key Recommendations for Action of the EU (Garske et al., 2020), as well as the establishment of the "EU Food Loss and Food Waste Platform." European countries such as Germany and Norway have elevated the fight against food waste to the level of national strategies. Germany has made reducing or avoiding food waste a national task in its National Strategy to Reduce Food Waste in 2019. Norwegian Government's 2017 Food Waste Reduction Agreement specifies that all actors in the food supply chain and the Government must take responsibility for food waste. Many countries, such as the USA, Argentina, South Korea, and Japan (Table 3), have elevated the fight against food waste to a national strategic level and introduced relevant laws and regulations.

**Table 3:** Anti-food waste strategy level documents of the world's leading countries and institutions

Countries (regions, institutions)	Laws and regulations and related documents	Date	Main content
EU	Circular economy package	2015	Provide a range of measures to reduce food waste
	EU guidelines on food donation	2017	The main focus is on how to regulate food conservation
	Waste framework directive	2018	Revisions revolve around increasing food conservation
	Developed by the EU platform on food losses and food waste	2019	Establishment of the "EU platform on food loss and food waste"
	European green deal	2020	Strategic objectives include "revision of EU food date marking rules, legal constraints on food waste."
	Food redistribution in the EU	2020	A comprehensive review of the legal and policy measures of the EU and its member states concerning food donation, such as food hygiene, labeling, liability, and taxation
UN	International agreement on world food security	1974	The concept of "food security" was introduced, regulating the relevant elements of food security
	Convention on biological diversity	1992	The regulations highlight the importance of food as a food resource for human survival and health and reflect the severe consequences of food waste on human development and the need for food conservation
	Rome declaration and plan of action	1996	Every person has the right to food and food security, and it recommends that governments specify in their domestic legislation the responsibility for food security in terms of poverty eradication, industrial development, increasing production, and reducing losses
	Think.Eat.Save	2013	It aims to highlight the environmental problems caused by food waste and calls for human beings to pay attention to food conservation
	The 2030 agenda for sustainable development	2015	Sets a sustainable development goal of halving global food losses in retail, consumption, production, and supply by 2030
	The Paris agreement	2016	Proposes to prioritize food security and hunger eradication
Germany	National strategy to reduce food waste	2019	Set targets to halve food waste at the retail and consumer levels and reduce food waste at the production and supply levels by 2030, and make the reduction or avoidance of food waste a national task
Norway	Agreement to reduce food waste	2017	It specifies that food producers, manufacturers, wholesalers, retailers, restaurants, households, and government departments must take responsibility for reducing food waste to promote food conservation and combat it
UK	National waste reduction strategy for England	2007	Emphasis on the economic and environmental impact of reducing food waste, with the main aim of reducing inter-household food waste
USA	Formal agreement among EPA, FDA, and USDA relative to cooperation and coordination on food loss and waste	2018	The agreement aims to improve coordination and communication between federal agencies to educate the American public better to reduce food loss and waste and, to some extent, promote the food conservation process
Argentina	National program to reduce food loss and waste	2019	It refines the regulations on food donations, the parties involved, the implementation process, etc.
Korea	Food waste reduction masterplan	1996	Regulations are in place for food waste recycling
Japan	The act on promotion of food loss and waste reduction	2019	Clarifies the Government's responsibility to avoid food waste

*EU* European Union, *UN* United Nations. *UK* United Kingdom of Great Britain and Northern Ireland, *USA* The United States of America, *EPA* Environmental Protection Agency, *FDA* Food and Drug Administration, *USDA* United States Department of Agriculture

Thrift and diligence have long been traditional virtues in China; however, with the development of the economy, waste has become more severe. The Party and the State have noticed this problem, and Table 4 summarizes the relevant policy documents on anti-food waste since Economic Reform and Opening. The initial period was primarily for food loss and waste of party and government organs. Since the 18th CPC National Congress, President Xi Jinping has made many speeches and instructions on the issues surrounding food and beverage waste. Furthermore, anti-food waste has risen to the level of national strategy. The introduction of this stage has also expanded from the initial opposition to grain waste to the opposition to food waste (Zhao, 2020). The main body of anti-food waste has been expanding, from the initial party and government organs to all walks of life.

**Table 4:** Documents related to the anti-food waste in China

Country	Laws and regulations and related documents	Date	Main content
China	Outline of the reform and development of China's food structure in the 1990s	1993	Adopt the necessary legislative and economic interventions to promote scientific and civilized food consumption and to oppose or discourage bad habits such as gorging and wastefulness
	Agricultural law	1993	It provides for the "promotion of food conservation and saving and the improvement of the nutritional structure of the people's food."
	Food problem in China	1996	Proposes three main measures to save food
	Several provisions on the party and government organs' efforts to exercise economy and stop extravagant and wasteful behavior	1997	It is strictly forbidden to use public funds to eat, drink or squander
	Circular on the work of central and state organs to conserve food and oppose food waste	2008	All departments and units must carry out extensive education and publicity on food conservation and anti-food waste
	National food security mid-term and long-term planning framework (2008–2020)	2008	Advocate scientific food conservation and curb mindless consumption of food and oil; establish civilized norms of "green dining and food conservation" in canteens, restaurants, and other catering establishments
	Circular on further strengthening food conservation and anti-waste efforts	2010	The basic guidelines for food conservation and anti-waste work in this period were established
	Circular economy development strategy and immediate action plan	2013	To promote economic and moderate consumption, oppose wastefulness, and carry out "anti-food waste actions
	Opinions on vigorous conservation against food waste	2014	Propose to promote the resourceful use of food waste
	Food law (draft for review)	2014	Chapter 5, entitled "Food Consumption and Conservation," covers food conservation and loss reduction in all stages of food storage, transportation, processing, and consumption and provides for a national system of penalties for reckless waste of food that causes severe food losses
	Measures for the management of catering industry (for Trial Implementation)	2014	Encourage enterprises to provide standardized dishes, develop optional set menus and provide small portions; establish a reminder system for frugal consumption; guide consumers to order the right amount of food before meals, take the initiative to help pack after meals, and give praise and rewards to consumers who save on meals; dispose of kitchen waste according to regulations; and prohibit the setting of minimum consumption
	Opinions on accelerating the construction of ecological civilization	2015	Foster a green lifestyle. Promote the concept of frugal and economical consumption. Catering companies, unit canteens, and households carry out anti-food waste actions on all fronts. Party and government organs and state-owned enterprises to take the lead in practicing diligence and frugality
	Several opinions on promoting the development of green catering	2018	It is required to practice the concept of green development and further improve the green catering standard system, service system construction, and green catering main construction
	Food security in China	2019	Promote food saving and loss reduction. Vigorously carry out publicity and education activities to enhance awareness of food care and conservation, curb unreasonable consumption demand and reduce "waste at the table."

Organized according to Zhao (2020)

#### Enactment of a single-line anti-food waste law

Currently, four countries in the world have introduced a single law with the primary goal of opposing food waste (Table 5) and consist of five laws concentrated in the European region. France was the first country to introduce an Anti-Food Waste Law. By comparing the anti-food waste laws of each country, the primary legal measures can be divided into punishment and encouragement. France, Spain, and China all have a legal policy based on punishment (Feng et al., 2022). In contrast, Italian law adheres to the principle of encouragement (Franco & Cicatiello, 2021). Regarding the specific content of the laws, France's Anti-Food Waste Law has explicit legal provisions for subjects in all parts of the food supply chain, with Italy's law primarily using incentives for food donations to reduce food waste. Spain's Anti-Food Waste Law requires all sectors of the entire food chain, from the primary or agricultural sector to the tertiary sector or retail and hospitality, to develop a reduction waste program. China's law focuses on food waste on the consumer side, and the main subjects are primarily catering service enterprises (Table S3).

**Table 5:** Comparison of single-line World Anti-Food Waste law

The Law	Country	Date	Main content	Type of policy	Food Chain
The anti-food waste act	France	2016	1. Provides for a waste hierarchy for food waste	Punishment oriented	The whole food chain
			2. Legal regulation of food production, sale, consumption, and related subjects		
			3. Emphasis on penalties for violations by food retailers		
The anti-food waste act	Italy	2016	1. Encourage donations using tax relief	Encouragement is the main focus	The whole food chain
			2. Simplify food donation procedures and provide more suitable conditions for food donations		
Circular economy and anti-waste act	France	2020	1. Prohibit businesses from destroying unsold non-food products	Punishment oriented	The whole food chain
			2. Increase the number of fines for food waste		
The anti-food waste act	Spain	2021	1. Even if the food is "low value," businesses should not waste or discard it at will	Punishment oriented	The whole food chain
			2. For shops over 400 square meters, there is an obligation to market foodstuffs that are still fit for consumption but poorly presented		
			3. Encourage the sale of seasonal, bulk-priced, locally produced foods that are not easily preserved for long periods		
PRC law on countering food waste	China	2021	Articles 27 to 30 set out the penalties for food waste	Punishment oriented	Concentrated on the consumer side

China's law is aimed at the main body of the restaurant business

We found that the focus on food waste reduction as an explicit national strategic goal and series of measures is mainly in developed countries, while developing countries are currently not paying enough attention to food waste reduction. The single law on food waste in China mainly focuses on the consumption end and pays less attention to the production end. Compared with developed countries, there are few incentive measures.

### Food donations and food banks

#### Regulating and encouraging food donations

Food donation is one of the most significant measures to solve today’s food waste (Busetti, 2019; Kinach et al., 2020; Sundin et al., 2022). Developed countries such as Europe and the USA have comprehensive laws on food donation, and explicit provisions for possible disputes and liabilities (Eriksson et al., 2015; González-Torre & Coque, 2016; Vlaholias et al., 2015). Some countries have tax deductions for companies or individuals who donate food. A summary of the current food donation laws worldwide shows that the incentives for food donation are generally the same in all countries (Table 6), with "escape clause + tax incentives" comprise the majority of tax incentives. These policies have given a strong impetus to the Act of food donation. The models of food donation can be divided into advocacy donation, represented by the US model, and compulsory donation, represented by France. The only Chinese law regarding food donations is Article 23^5^ of the Anti-Food Waste Law, and The Chinese Civil Code.^6^ The Law on Public Welfare Donations make only vague references to donations of goods of all property types.

**Table 6:** Comparison of relevant national (regional, institutional) laws and regulations on food donation

Country	Related documents	Time	Specific measures	Type of system	Donation mode
USA	Bill Emerson good Samaritan food donation act	1996	Clarify that food donors and non-profit organizations that distribute food are not legally liable for this	Disclaimers	Advocacy donation
	Federal food donation act of 2008	2008	The bill requires that "all [federal] contracts for the provision of services or the sale of food in the United States that exceed $25,000 shall contain a clause	Tax system	
	Protecting Americans from tax hikes (PATH) act	2015	The Act makes all businesses eligible for a tax deduction for donations that meet specific eligibility criteria	Tax system	
Italy	Good Samaritan law	2003	The bill is intended to promote the exemption of food donors from liability	Disclaimers	Advocacy donation
	Law No. 166 on the donation and distribution of food and pharmaceutical products for purposes of social solidarity and food waste prevention	2016	The bill encourages expanding the range of food donors and tax benefits for food donations	Tax system	
Argentina	The food donation act	2019	Refines the rules on food donations, the parties involved, and the implementation process, and exempts food donors from legal liability for expired food, etc.	Disclaimers	Advocacy donation
Czech Republic	Act no. 110/1997 coll., on food and tobacco products	2017	Mandatory food donation for supermarkets with a more than 400 square meters business area		Compulsory donation
	The food grain taxation system	2017	Supermarkets are no longer required to pay 5% VAT on donated food	Tax system	
France	Food waste reduction bill	2016	Food sellers deemed to be hypermarkets must donate food that does not affect consumption 48 h before the final day of arrival of the food for consumption. The Act also provides incentives in the form of tax credits	Tax system	Compulsory donation
China	Civil Code	2020	Article 662: "The donor shall not be liable for any defects in the property gifted	Disclaimers	Advocacy donation
	Public benefit endowment act	1999	Chapter IV preferential measures provide for donating property and materials for public welfare; enterprises can enjoy the federal tax benefits	Tax system	

*USA* The United States of America

#### Food bank

Most food donations are distributed through food banks as a single organization. Food banks first originated in the USA to address the coexistence of waste and hunger. The world's first food bank was established in the United States in 19,676.^7^ After more than half a century of development, there are now nearly 1,000 food banks worldwide and at least 400 charities worldwide. The Global Food Bank Network is currently the largest food bank organization, connecting and empowering food banks affiliated with regional and national food bank networks in more than 40 countries.^8^ The main item distributed by food banks is food, but the USA, Canada, and other areas also distribute money or paraphernalia such as food stamps (Table 7).There are two main sources of donations to food banks, one is social donations and the other is government funding (Lambie-Mumford 2013; Warshawsky, 2011; Yadlowski & Theriault, 1998). The governments of USA and Canada provide funds to food banks in order to support their food purchases, and the US Department of Agriculture cooperates with food banks to promote certain projects. In terms of the role played by food banks, they play a significant role in assisting the disadvantaged and reducing food waste. Due to the traditional attitude of the Chinese, many needy groups will not go to food banks to receive help due to pride and dignity. Therefore, the Oasis Food Bank has innovated and developed the world's first online pilot food bank, where users in need can get help from food banks by placing orders for online delivery.

**Table 7:** Comparison of food banks in major countries around the world

Country	USA	Canada	SA	UK	Spain	China
Type of donation	Food and money	Food and money	Food	Food	Food	Food
Established	1966	1981	2009	1986	1987	2014
Donors	Private and Government	Private	All organizations	Private and government	Private and government	Private
Government funding	State and local governments	Local government	State	None	None	None
Main beneficiaries	Low-income households	Children and sick people	People who are homeless or malnourished	Low-income households	Low-income households	Various groups of people in need
Total food banks	> 200	< 500	5	250	55	< 10
Number of beneficiaries	> 37,000,000	> 800,000	> 300,000	> 120,000	> 1,000,000	> 1,100,000
Networks or organizations to join	Feeding America; Global food bank network	Canadian food bank organization; global food bank network	Food bank of South Africa	European federation of food banks	Spanish federation of food banks	Global food bank network; China food bank network
Number of food recalls	Food waste reduction of £4.7 billion (2020)	Approx. 12,700t (2020)	12013t (2021)	27000t (2021)	Approx. 10,750t (2015)	Approx. 260.2t (2020)

*UK* United Kingdom of Great Britain and Northern Ireland, *USA* The United States of America, *SA* South Africa

In general, food donation plays an important role in reducing food waste, and developed countries are quite complete in terms of specific requirements and exemption clauses for donations. As a major distributor of food donations, food banks are growing rapidly around the world and playing an important role in reducing food waste. China's food banks are still in the early stages of development, but their online distribution method is worth promoting.

### Food recovery

Food waste recycling is also an important measure to reduce food waste. How to develop an effective food recycling framework has attracted wide attention from countries around the world. Korea has adopted a strict food waste charging policy for food waste. The Korean government has executed three methods of charging for food waste, which include Radio Frequency Identification (RFID) cards, prepaid garbage bags, and barcode management systems to reduce food waste. According to a study in Korea, the adoption of a measured food waste fee has reduced the amount of food waste generated in Korea by 300 tons per day. The US Environmental Protection Agency provides a food recovery hierarchy chart to address food waste, with favorite and least favorite options from the top to the bottom of the pyramid. These methods include "Source Reduction", "Feeding the Hungry", "Feeding Animals", "Industrial Use", "Vomposting" and "Landfill/Incineration". The EU has also made detailed regulations on the recycling hierarchy of food waste (Table S4). However, China has only made simple regulations based on the 3R (Reducing, Reusing, and Recycling) principle, and does not currently have a clear food recycling hierarchy. Although the policy of waste separation has been implemented, the food waste generated is not recycled sufficiently, and much of it is wasted. Developing countries, such as China, should focus on setting reasonable levels of food recovery and refining implementation measures in order to minimize unnecessary waste and greenhouse gas emissions, thereby contributing to the achievement of sustainable development goals.

### Anti-food waste social governance

Food waste exists in the entire supply chain of food production and involves a variety of subjects. Therefore, to solve food waste, we cannot rely on the power of the Government alone but need the participation of multiple social subjects to solve the problems related to food waste.

#### Multiple subjects participation

Germany launched a "Too Good for Bins!" competition in 2012, which is for all companies, startups, tours, farms, research institutions, NGOs, and individuals to participate in a program of ideas and actions to reduce food waste. In the United States, the US Food Waste Challenge was launched in 2013 by the Agriculture and Environmental Protection Agency, calling on entities throughout the food chain, including farms, Agri-processors, food manufacturers, grocery stores, and tours. Norway established Matvett, a public interest organization that brings together food manufacturers, wholesalers, supermarkets, and consumers to reduce food waste by 12 percent between 2010 and 2015. In 2020, core members of the Norwegian food service industry launched the “Food Waste Reduction 2020" program”, which mobilized 50 percent of industry participants to join the effort.

#### Civic education

Household waste is the primary source of food waste (Dou & Toth, 2021); therefore, strengthening public education is a significant way to reduce waste. Italy, France, and other European countries have introduced food waste reduction into the classroom and used educational measures to raise students' awareness against food waste (Hou et al., 2018). Japan has enacted the Basic Law of Food Education to educate all citizens to value food and reduce waste. Germany has organized an annual nationwide "Save Food in Germany" awareness week since 2020. China also had several activities related to anti-waste including the so-called “Clean Plate 2.0 campaign”, to raise public awareness of the issue, cultivate thrifty habits and foster a social environment where waste is less acceptable and thriftiness is good, to prevent food waste.

### Labeling of food dates

A study by the European Commission in 2018 showed that up to 10% of the 88 million tons of food waste generated annually in the EU is related to the date label (European Commission. Directorate General for Health and Food Safety. et al., 2018). Consumers determine whether food is ready for consumption by the expiration date on the food packaging. Globally, the terms used for food dates appear on food packaging (Table 8) and include "expired", "sell by", and "best before". This greatly confuses consumers and increases food waste; therefore, improving the consistency and clarity of food date labels can effectively reduce waste. Most countries have adopted laws and regulations to clarify the use of food label dates and circumstances (Weis et al., 2021). The US Department of Agriculture (USDA) unified food label dates as "Best if used by" in order to solve the confusion of food label dates and the waste caused by the public misunderstandings about food shelf life. At the same time, the definition also has actively strengthened public knowledge. In Denmark and the UK, social organizations actively cooperate with supermarkets in order to educate people about food shelf life. In China, the shelf life of food is uniformly marked on the date of labeling, but it is a broad concept of shelf life. Date labelling and shelf life standards for food are not specified in the law, resulting in the waste of much foods that could have been recycled and donated. There is a lack of relevant education at the social level, resulting in more food being wasted by Chinese families due to date.

**Table 8:** Comparison of the content of food date labels

Country (institution)	Time	Laws or documents	Specifics
EU	2011	Regulation (EU) No. 1169/2011	Clarifies the different situations and ways of labeling "minimum durability date," "use by" date, and "freeze by" date on food labels
USA	2017	USDA regulations	The date on food labels will be standardized as "best if used by" and required to be used by egg, meat, and dairy producers
Italy	2016	Article 2 of law no. 166	The distinction between "best before data" and "expiration date."
France	2016	Circular economy law	The distinction between "best before" and "longest use by" dates should be clarified and made clear to consumers on the label that some foods can be consumed beyond their best before dates
China	2015	Food safety law	The concept of "shelf life of food" is used in the food date labeling system and is defined as "the period during which the food retains its quality under the storage conditions indicated."

*EU* European Union, *USA* The United States of America

### Data collection on food waste

Strengthening international cooperation and scientific research projects in food waste data collection has a very important role to play in reducing food waste. One of the critical shortcomings of food waste research is the shortage of food waste data (Fabi et al., 2021; Nicastro & Carillo, 2021b). Internationally, FAO has been collecting food waste data and has published in The State of Food Security and Nutrition in the World and Food Loss and Waste annually in recent years. The World Food Waste Index published by the United Nations Environment Programme (UNEP) is in collaboration with WRAP. The Report is the most authoritative and complete food waste data in the world. The UNEP and WRAP released the World Food Waste Index report and is the most authoritative and complete food waste data in the world. The EU makes explicit reference in the document to the establishment of a food loss and waste database and the EU common food waste measurement standards. The USA has also started collecting food waste data for quite a while, with the Department of Agriculture as the main body and various companies cooperating to collect relevant food waste data (Table 9). China's food waste data collection is abridged, and the majority of data used is China's food waste data collection which is extremely short. The database most commonly used is CHNS database (Min et al., 2021; Qi et al., 2021) and the China Restaurant Waste Report (Xu et al., 2020).

**Table 9:** Comparison of significant food waste databases

Country (institution)	Databases or documents	Data collection methods	Key data content
FAO	The state of food security and nutrition in the world	The FAO food balance sheets are produced by setting up fixed observation points around the world to collect data from all parts of the food supply chain, from which the scale of world food loss and waste can be estimated	Mainly global data on food loss and waste, the database contains data by country, but accuracy is to be considered
	Food loss and waste database		
	Food Loss and Waste		
UN	World food waste index report	Major countries were selected as a sample to collect data on waste at various stages of the supply chain and to estimate the scale of global food waste	The content of the food waste data, which is currently more credible and complete globally, is available
EU	EU centre for food loss and waste prevention	Launch of the FOODRUS survey to investigate the amount of food waste at all stages from farm to fork	It contains data on food waste in most EU member states, but the data is slow to be updated and is currently stuck in 2015
USA	Food waste in America report	Data on food waste is collected by the ministry of agriculture, with the cooperation of selected companies across the country	Overall food waste situation in the U.S. and household waste, latest data available for 2020
China	CHNS database	Data collection is carried out for household and catering waste in the form of weighing and meal review methods	The volume of data is small, mainly for household food waste, while only seven issues contain food waste information, the most recent being 2009
	China urban catering waste report		A significant waste survey in China's restaurant industry, with data available for 2017 only

*FAO* Food and Agriculture Organization of the United Nations**,** *UN* United Nations, *EU* European Union, *USA* The United States of America

## Discussion

### A comparison of global food waste policy experiences

Food waste management has implications in several policy areas including sustainable resource management, climate change, energy, biodiversity, habitat protection, agriculture and soil protection (Garcia-Herrero et al., 2018; Secondi et al., 2015). Policy-driven is the main measure to reduce food waste (Hamilton & Richards, 2019; Tang et al., 2022; Thyberg & Tonjes, 2016; Urrutia et al., 2019). One potential regulatory instrument is the review and elimination of unnecessary food safety standards that lead to high food waste rates. In comparison to fiscal and economic incentives, well-defined regulations seem to be a more effective tool to combat household food waste generation (Chalak et al., 2016). Following the introduction of the UN Sustainable Development Goals in 2015, several countries have elevated food waste reduction to the level of national strategies (Mourad, 2016). However, through our analysis, we found that most of them belong to developed countries at present, and developing countries are less concerned about food waste due to the existence of hunger and other phenomena.

Food sharing and food banks are a good solution to the problem of excess food and have a significant effect in reducing food waste (Capodistrias et al., 2022; Falcone & Imbert, 2017; Makov et al., 2020; Morone et al., 2018). Countries mainly use economic incentives for food banks, which include tax incentives and subsidies. Economic incentives aim to reduce food waste through costs or other market signals (Driesen, 2006). Exemption clauses are also important in the area of surplus food donations, and China's current legal policies are very lacking in this area, resulting in the current slow development of food banks.

The prevention and recycling of food waste contribute to a circular economy due to improvements in resource efficiency and energy recovery (Fujii & Kondo, 2018; Lin et al., 2014). Studies have found that sound food recycling policies can contribute to sustainable development, especially for developing countries (Fogarty et al., 2021; Fujii & Kondo, 2018; Lu et al., 2022a; Sarker et al., 2022). Our results found that Europe and the United States have a more rational food recovery structure with detailed rules for reducing food waste. In this regard, developing countries such as China need to learn from this, which currently has a shi'dian food recovery hierarchy in some cities.

Reducing food waste requires the participation of the entire community, information campaigns present one of the most widespreadtools used for food waste prevention and reduction (Priefer et al., 2016). Increased education of the population to reduce food waste is also very essential, such as cooking classes to help reduce household food waste (Mondéjar-Jiménez et al., 2016), and in some countries, food education has been added to the curriculum. Interventions that place a household's food waste level in relation to societal averages or a socially endorsed goal (bench marking) result in stronger norm activation (Porpino et al., 2016). Therefore, a culture of reducing food waste should be developed in the whole society, and there are still sectoral shortcomings in China in this regard.

Date labelling on packages is a key instrument of food policy, situated between production, retailing and consumption (Milne, 2012). Education about the meaning of date labels and efforts to improve the acceptability of imperfect foods (e.g., foods that are not fresh, unattractive, or close to their expiration dates) are also important measures to reduce food waste (Milne, 2012; Newsome et al., 2014). To prevent consumer confusion about expiration dates, a huge potential for reducing food waste lies in optimizing the labeling of prepackaged foods. There are a number of labeling technology innovations that can be adopted to reduce food waste.

Due to uniform food waste measurement standards and methods, there is less food waste data in the world, and it is especially difficult to collect food waste data for developing countries. In the future, it is necessary to further strengthen international cooperation to obtain food waste data in various aspects, to further measure the resource and environmental effects caused by food waste around the world, and to promote the process of global sustainable development.

We have compared current policies on food waste in countries around the world; however, there are many factors that influence the food waste phenomenon, and its policy development needs to take into account with national conditions in many ways (Morone et al., 2019a). For example, multinational companies and cultural industries can affect food waste, and in some countries, there are "food deserts" where hunger and waste coexist (Dagar et al., 2021). Therefore, we need to strengthen international cooperation and develop policies to reduce food waste according to local conditions.

### Experience and inspiration for China

As the world’s largest emerging economy, China’s food security is of special concern, particularly because while it contains approximately 20% of the world’s population, China only encompasses 7% of the world’s arable land. Through the previous comparison of global food waste reduction policies, we found that there are still some problems in the process of policy development and implementation in China. Combining the current development situation in China and learning from foreign advanced experience, we propose the following recommendations to promote the implementation of China's food waste reduction policy, help “carbon neutral &carbon peak”, and to achieve sustainable development.

#### Improve the legal system against food waste and refine the norms for implementing legal policies

China has enacted several anti-food waste policies, and in 2021 introduced the Anti-Food Waste Law to elevates food waste to a national strategic level. However, laws such as the Anti-Food Waste Law do not specify how to enforce the law, define anti-food waste behavior, and punishment or reward. Meanwhile, China's laws and policies are primarily aimed at consumer-level waste, with little design for other stages of the food supply chain. First, the rules for implementing the law should be further improved. According to the specific situation in China and the international experience, specific measures and policies can be issued by localities, considering both penalties and rewards, stipulating wasteful behaviors of various subjects in the food supply chain, and specific implementation methods such as rewards and fines. Second, the collaboration among various departments should be strengthened to promote the implementation of relevant laws and policies jointly.

#### Formulate legal policies related to food donation and regulate the development of food banks

Food donation plays an essential role in addressing the issue of food waste and hunger at an international level. Article 23 of the Anti-Food Waste Law introduces the concept of food donation at the legislative level in China. China's food banks are in the early stages of development, and a single law cannot satisfy the establishment of China's food donation system and the development of food banks. It is recommended that food donation-related legislation should be introduced as soon as possible and that special legislation on food donation be considered, which is a single law regulating food donation and specifies the donation process and exemption measures of the law. Increase tax incentives, propose to revise relevant tax regulations, introduce relevant tax relief measures, and encourage enterprises, and individuals to donate food. The government needs to cooperate with non-profit organizations to build a food donation platform and strengthen the guidance for food banks to establish a food bank system with Chinese characteristics.

#### Establish a multi-level food recovery system to enhance the efficiency of food recovery and reuse

First, focus on multiple measures to reduce waste at the source. Increase investments in science and technology to reduce food loss and waste in the post-harvest grain and food supply chain. In accordance with the South Korean garbage metering and charging model, a nationwide waste metering and charging pilot will need to be carried out. Domestic waste will be charged step by step, and relevant expired food donations will be collected. Moreover, all these steps will need to be taken in order to reduce food waste at the source. The next step is to recycle food. Following the international experience, food that is not fit for human consumption should be fed to animals. The waste separation system should be improved. Following the example of Germany, catering enterprises should install oil–water separation equipment, and provide waste oil, which can be used as industrial fuel, compost or biogas to generate electricity. Finally, the remaining food waste should be disposed of in a uniform "landfill or incinerator."

#### Engaging diverse social actors to make anti-food waste conscious

Food waste involves all subjects in the food supply chain and requires the concerted efforts of all community sectors to solve this problem. First, we should strengthen the publicity of green consumption, create a green living culture in the community, establish positive and negative models, and increase rewards and punishments. In addition, we also should strengthen publicity and education, carry out anti-food waste education in all classrooms, and guide social subjects to spontaneously carry out anti-food waste work. Finally, we should learn from overseas experience and carry out more a government-funded anti-food waste campaign attracting the participation of various social organizations.

#### Strengthen research projects and collect food waste data on multiple levels

The lack of food waste data is a major shortcoming of China's food waste prevention and control. A comprehensive collection of food waste data will help us to understand the food waste problem scientifically and systematically so that we can better implement relevant measures to solve the issue. It is recommended to strengthen the establishment of scientific research projects on food waste, integrate multidisciplinary research on food waste, and provide academic support for policies. Meanwhile, it is also recommended to strengthen the collection of food waste data, set up nationwide food waste data collection points, and mobilize universities, research institutes, local governments, and companies to collaborate and collect food waste data in a comprehensive and multidisciplinary manner in order to provide data support for the fight against food waste.

## Conclusion

We compared global food waste reduction policies in terms of legislation, food donation, food recovery, social engagement, date labeling and data collection. We found that most countries have elevated food waste reduction to the level of a national strategy, while some have enacted one-line laws against food waste. Food donations and food banks are effective measures to reduce food waste, with the main policy being "exemptions + tax incentives". Because hunger is still a problem in some developing countries, food donation policies have been slow to spread in developing countries.

A multi-level food recovery system not only reduces unnecessary food waste, but also facilitates the development of a circular economy, reduces unnecessary greenhouse gas emissions, and thus promotes the achievement of sustainable development goals. Household food waste is a major contributor to the waste phenomenon, and increased education of residents, such as shelf life and cooking knowledge, is also an important means of reducing food waste. Strengthening international cooperation by collecting food waste data in a comprehensive manner is also an important goal for future research.

Our study also found that food waste policy development requires consideration of multiple factors, and the measurement of implementation effectiveness during implementation, especially for developing countries, is an important issue. Food waste is predicted to increase in the upcoming years as low- and middle-income countries become more wealthy (Barrera & Hertel, 2021; Gil, 2020). Most countries have policies in place to combat food waste, and these policies have had some effect. Developed countries are doing better at the level of legislation, leading social participation, and food donation. However, China's current anti-food waste policy is in its infancy and still has shortcomings.

China can improve its food waste policy in the future by refining legislation, guiding the development of food banks, promoting social governance, and strengthening scientific research projects. This will reduce food waste, reduce greenhouse gas emissions, and reduce the loss of resources and environment caused by food waste, thus advancing China's sustainable development process and contributing to the achievement of the UN 2030 Sustainable Development Goals.

In addition, establishing criteria to evaluate the actual effectiveness of different policies in order to develop the best measures against food waste is key to future anti-food waste policy research. The evaluation of policy formulation and implementation effects in developing countries will also be a focus of future country studies. In our future research, we will further assess the implementation effects of China's food waste policy and its impact on resource environment and sustainable development.

## Supplementary Information

Below is the link to the electronic supplementary material.

Supplementary file1 (DOCX 21 KB)

Supplementary file2 (XLSX 16 KB)

## Data Availability

This article is not applicable.

## Footnotes

1 Commonly referred to internationally as the food system.

2 The State of Food Security and Nutrition in the World 2019, published by the Food and Agriculture Organization of the United Nations.

3 In its 2019 report, the EAT-lancet Commission states that total food waste and biodiversity loss of 1043E/MSY (Extinctions Per Million Species-years) by 2050, with productivity and dietary structure unchanged, and that halving food waste could bring biodiversity if food waste were halved, this would reduce biodiversity loss to 684E/MSY.

4 SDG 12.3: "By 2030, halve per capita global food waste at the retail and significantly impacts food losses along production and supply chains, including post-harvest losses".

5
*Article 23:* The government should guide food production and operators, in the premise of ensuring food safety to the relevant social organizations, welfare agencies, relief agencies, and other organizations or individuals to donate food.

6 *Article 662: *A donor is not liable for any defect of the gifted property. Where a gift is subject to an obligation, if the gifted property has defects, the donor shall, to the extent of the attached obligation, bear the same liabilities as a seller.

7 The world's first food bank—St. Mary's Food Bank Alliance in Arizona, USA, was built in 1967.

8 *source:* https://worldfoodbank.org/
