# Supplementary material for: The status of the global food waste mitigation policies: experience and inspiration for China
Source: Environ Dev Sustain. 2023 Apr 5:1–29. Online ahead of print. doi: 10.1007/s10668-023-03132-0 (PMC10073629; doi:10.1007/s10668-023-03132-0)
Supplement: Supplementary file 1 — Supplementary file1 (DOCX 21 KB) [file 10668_2023_3132_MOESM1_ESM.docx]

Table S1 Definitions of food waste by leading organizations and academics

| Author /Institution | Data | Source | Concept definition |
| --- | --- | --- | --- |
| China | 2021 | *PRC law on countering food waste* | It is the failure to use food that is safe for consumption or drinking in a reasonable manner for its functional purpose, including disposal, reduction in quantity or quality of food due to unreasonable use, etc. |
| UNEP | 2021 | *Food Waste Index*  *Report 2021* | "Food waste" is defined as food and associated non-edible parts removed from the human food supply chain. |
| Zhang *et al*. | 2019 | *Journal of Natural Resources* | Food waste mainly refers to the discarding of the edible portion of food after harvest due to the irrational use of food. |
| Thyberg *et al*. | 2016 | *Resources, Conservation*  *and Recycling* | Food that was originally produced for human consumption but has since been discarded or is not consumed by humans. Includes food that has spoiled before disposal and food that is still edible after it has been thrown away. |
| USEPA | 2014 | *U.S. Environmental Protection Agency* | Food waste is defined as unconsumed food and food preparation waste in residential, commercial, and institutional settings. It does not include pre-consumer food waste arising from the food manufacturing and packaging process. |
| USDA | 2014 | *United States Department of Agriculture* | Food waste is a subset of food loss and occurs when an edible food item is not consumed. The only food that is still edible at the time of disposal is considered to be wasted. |
| EU | 2014 | *European Commission* | Food waste is defined as food lost from the food supply chain (including inedible portions), excluding food diverted to material uses such as biological products, animal feed, or food used for redistribution. |
| FAO | 2011 | *Global food losses and food waste* | The loss of food that occurs at the end of the food chain (retail and final consumption) is known as 'food waste. |
| Stuart | 2009 | *Waste: Uncovering the Global Food Scandal* | Includes edible materials intentionally fed to animals or food processing by-products diverted from human food. |
| Smil *et al*. | 2004 | *Environmental Sciences* | This includes over-nutrition, the gap between the energy value of food consumed per capita, and the energy value of food required per capita. |

Abbreviations:UNEP, United Nations Environment Programme;USEPA, U.S. Environmental Protection Agency;USDA, United States Department of Agriculture;EU, European Union;FAO, Food and Agriculture Organization.

Table S2 Comparison of the main measures of food waste

| Measurement methods | Main content | Advantages | Disadvantages | Related Research |
| --- | --- | --- | --- | --- |
| FAO Material Flow Model | The food supply chain was divided into five segments: production, post-harvest handling, storage, processing, distribution, and consumption. The data from the FAO food balance sheet and the waste ratio for each segment were then used to calculate the amount of food loss and waste. | (1) Facilitates cross-country comparisons  (2) Facilitates the calculation of food waste at all stages of the food supply chain.  (3) Flexibility to convert wasted quantities into units of heat for other studies. | (1) The data are more sketchy and the results obtained are more inaccurate.  (2) There are currently fewer data available internationally. | Kummu *et al*.  Hu *et al*. |
| Research method | Food waste surveys are conducted in one or more areas, at one or more points in the food supply chain, and include a variety of methods such as bookkeeping, archaeological methods, weighing, and dietary reviews. | (1) The data obtained reflects a more accurate picture.  (2) Access to first-hand data. | (1) It is time-consuming and requires a lot of money.  (2) The research process is vulnerable to interference from the researcher or other factors. | Harrison *et al*.  Koh WHO  Quested *et al*.  Lu *et al*.  Gao *et al*. |
| Literature Review Method | To make a judgment on the state of food waste by consolidating the relevant research literature. | (1) Save time and effort.  (2) More data is obtained. | (1) The authenticity and reliability of the data cannot be guaranteed. | D.L. *et al*.  Scott Kantor *et al*.  Monier *et al*. |

Table S3 Overview of the penalty conditions and measures of the Anti-food Waste Law

| Targeted actors | Examples | Penalty conditions | Penalty measures |
| --- | --- | --- | --- |
| Catering service providers | Restaurants, snack bars, beverage shops,  etc | Fail to actively remind consumers to prevent food waste  Mislead consumers to order excessive food and cause evident waste | **Warning:** take corrective action and be warned  **Warning:** take corrective action and be warned  **Fine:** if the violator refuses to take corrective action, they shall be fined between 1,000 and 10,000 Yuan. |
| Food producers or dealers | Food production and processing enterprises, food retailers, catering industry, canteens, etc | Cause serious food waste in food production and trade | **Fine:** take corrective action, and if the violator refuses to take corrective action, they  shall be fined between 5,000 and 50,000 Yuan |
| Entities with canteens | Schools, hospitals, enterprises, etc | Fail to formulate or implement measures to prevent food waste | **Warning:** take corrective action and be warned |
| Radio stations, TV stations or  network audios, and video service providers | Radio stations, TV stations or network audios, and video service providers | Produce, release, or disseminate programs or audio and video information advocating excessive food consumption and gluttony | **Warning:** take corrective action and be warned  **Fine:** if the violator refuses to take corrective action or the circumstances are serious, they shall be fined between 10,000 and 100,000 Yuan  Business suspension: and may be ordered to suspend relevant business or cease business operation for an overhaul  Other sanctions: the directly responsible executive in charge and other directly liable persons shall be subject to legal liability by the law |

Source: Feng(2022).

Table S4 Comparison of Food Recall Hierarchy in China, USA, and Europe

| Country | Food recovery hierarchy |
| --- | --- |
| USA | Source Reduction |
|  | Feeding the Hungry |
|  | Feeding Animals |
|  | Industrial Use |
|  | Composting |
|  | Landfill/Incineration |
| EU | Preventing waste |
|  | (Preparation) Reuse |
|  | Recirculation |
|  | Recycling |
|  | Disposal (including landfills and incineration where energy cannot be recovered) |
| China | Reduction (mainly food loss) |
|  | Reuse (food donation) |
|  | Resourceization (composting, biogas, power generation) |

Abbreviations:USA, United States;EU, European Union.

**Reference:**

Buzby, Jean C., Hodan F,Jeffrey H(2014). The Estimated Amount, Value, and Calories of Postharvest Food Losses at the Retail and Consumer Levels in the United States.US：U.S. Department of Agriculture.

Feng Y, Marek C, Tosun J (2022) Fighting Food Waste by Law: Making Sense of the Chinese Approach. J Consum Policy 45:457–479.https://doi.org/10.1007/s10603-022-09519-2

Gao L, Cheng S, Cao X, Zhang D,Liu Y,Wang L (2015) Review of Food Loss and Waste Research and Its Prospects. Journal of Natural Resources 30:523–536. <https://doi.org/10.11849/zrzyxb.2015.03.015>

Harrison GG, Rathje WL, Hughes WW (1975) Food waste behavior in an urban population. Journal of Nutrition Education 7:13–16. <https://doi.org/10.1016/S0022-3182(75)80062-8>

Hu Y, Zhou Y, Han Y,Xu Z(2013) Resources and Economic Effects Analysis of Reducing Food Waste. China Population,Resources and Environment 23:150–155.

Huang J, Nie F (2016) Research Advancement of Food Loss and Waste Studies.Food and Nutrition in China Vol.22:43–47.

Kummu M, Moel H de, Porkka M, Siebert S, Varis O, Ward P (2012) Lost food, wasted resources: Global food supply chain losses and their impacts on freshwater, cropland, and fertiliser use. Science of The Total Environment 438:477–489. <https://doi.org/10.1016/j.scitotenv.2012.08.092>

Lu S, Cheng G, Li T,Xue L,Huang J,Liu G (2022) Quantifying supply chain food loss in China with primary data: A large-scale, field-survey based analysis for staple food, vegetables, and fruits. Resources, Conservation and Recycling 177:106006. <https://doi.org/10.1016/j.resconrec.2021.106006>

Martindale W (2010) Waste: uncovering the global food scandal. International Journal of Sustainable Engineering 3:144–145. <https://doi.org/10.1080/19397030903573317>

Smil V (2004) Improving Efficiency and Reducing Waste in Our Food System. Environmental Sciences 1:17–26. <https://doi.org/10.1076/evms.1.1.17.23766>

Thyberg KL, Tonjes DJ (2016) Drivers of food waste and their implications for sustainable policy development. Resources, Conservation and Recycling 106:110–123. <https://doi.org/10.1016/j.resconrec.2015.11.016>
